# Books and Magazines

**Published:** 1905-07

**Authors:** 


### Books and Magazines.

---

Saunders' Pocket Medical Formulary. By William M. Powell, M. D., author of "Essentials of Diseases of Children"; Member of Philadelphia Pathological Society. Containing 1831 formulas from the best known authorities. With an Appendix containing Posological Table, Formulas and Doses for Hypodermic Medication, Poisons and their Antidotes, Diameters of the Female Pelvis and Fetal Head, Obstetrical Table, Diet-list, Materials and Drugs used in Antiseptic Surgery, Treatment of Asphyxia from Drowning, Surgical Remembrancer, Tables of Incompatibles, Eruptive Fevers, etc., etc. *Seventh Edition, Revised.* In flexible morocco, with side index, wallet, and flap. $1.75 net. Philadelphia and London, W. B. Saunders & Co., 1905.

When a work has reached its seventh edition there can be no doubt of its practical usefulness. And it is not at all surprising to us that Saunders' Pocket Medical Formulary should have attained such popularity, for we know of no similar work containing so much useful, practical and accurate information in so small a compass. In this new seventh edition there have been added over 460 new and valuable formulas, selected from the works and private practices of the best authorities. The editor has shown rare discretion in the elimination of many obsolete formulas, inserting in their place new and better ones, embodying a large number of approved new remedies. In its new edition this Formulary is thoroughly representative of the most recent therapeutic methods, and its convenient size and mechanical get-up make it the most desirable work of its kind on the market.

---

A Text-Book on the Practice of Gynecology. For Practitioners and Students. By W. Easterly Ashton, M. D., LL. D., Fellow of the Gynecologic Society; Professor of Gynecology in the Medico-Chirurgical College of Philadelphia. Octavo volume of 1079 pages, containing 1046 new and entirely original line drawings. Philadelphia and London: W. B. Saunders & Co., 1905. Cloth, $6.50 net; Half Morocco, $7.50 net.

Dr. Ashton's practice of Gynecology is a new departure in medical text-book making. The author takes up each procedure step by step, the student being led from one step to another just as in studying any non-medical subject. Nothing is assumed, Dr.

Ashton in every instance not only telling what should be done, but also precisely *how to do it.* All the methods and details of technic described have been thoroughly tested by the author himself, so as to assure their value and accuracy. A very commendable feature is the departure from the old routine method of devoting a general chapter to physical examination. In place of this the author presents the examination of each organ separately before describing its diseases, thus greatly aiding the student in familiarizing himself with the technic. A distinctly original feature consists in the line drawings made especially for this work under the author's personal supervision from actual apparatus, living models, dissections on the cadaver, and from the operative technics of other authors. There are ten hundred and forty-six of these illustrations, showing the procedures and operations without obscuring their purpose by unnecessary anatomic surroundings. Definite and precise instructions are given regarding the preservation of specimens of morbid tissues and secretions, and their delivery in good condition to the pathologist. The fore part of the work, dealing with antiseptic technic, shows great care in its preparation, Dr. Ashton wisely describing only those methods which he employs in his own practice, in order that the reader may have a clear and definite conception of the subject. Very special attention has been given to the consideration of visceral injuries, and we know of no other work on gynecology or general surgery discussing this important subject with the same amount of detail. This is decidedly a work for the general practitioner as well as for the student; and a good one.

---

MALARIA, INFLUENZA, AND DENGUE. By Dr. J. Mannaberg, of Vienna, and Dr. O. Leichtenstern, of Cologne. Entire volume edited, with additions, by Ronald Ross, F. R. C. S., F. R. S., Professor of Tropical Medicine, University of Liverpool; J. W. W. Stephens, M. D., D. P. H., Walter Myers, Lecturer in Tropical Medicine, University of Liverpool; and Albert S. Grunbaum, F. R. C. P., Professor of Experimental Medicine, University of Liverpool. Octavo volume of 769 pages, fully illustrated, including eight full-page plates. Philadelphia and London: W. B. Saunders & Co., 1905. Cloth, $5.00 net; Half Morocco, $6.00 net.

This new volume in Saunders' American Edition of Nothnagel's Practice represents the latest work on the subjects of which it treats. And more than that: It is the undisputed authority on these subjects. For this American edition Dr. Ross has made additions to the article on Malaria, so many discoveries having been made since the appearance of the original article. The articles on the Mosquito and its various relations to Malaria come from the authoritative pen of Dr. J. W. W. Stephens, of Liverpool. The Influenza and Dengue sections are equally well written. The untiring labor of the editors in preparing this work for the English-speaking market is evidenced on almost every page by the lengthy and valuable editorial interpolations. This is the tenth volume in the series, and the eleventh one (that dealing with Diseases of the Kidneys and Spleen and with Hemorrhagic Diseases) is promised very soon. When the series is completed it will undoubtedly form the best practice of medicine in existence.

---

Modern Methods of Treatment—The Open-Air Treatment of Pulmonary Tuberculosis. By F. W. Burton-Fanning, M. D., Consulting Physician to the Norfolk and Norwich Sospital; Honorary Visiting Physician to the Kelling Open-Air Sanatorium. W. T. Keener & C., Chicago, 1905. 175 pages. Cloth, $1.75.

Consumption is a house disease, a disease of "civilization." The medical profession has been a long time finding out the rationale and value of out-of-doors treatment; of putting the consumptive in touch with nature; in natural instead of artificial surroundings. Better late than never. It's the thing, and the only thing that "cures." Drugs are not in it any longer. This little book is a practical guide to the modern method of managing pulmonary tuberculosis.

---

Atlas and Text-Book of Topographic and Applied Anatomy. By Prof. Dr. O. Schultze, of Wurzburg. Edited, with additions, by George D. Stewart, M. D., Professor of Anatomy and Clinical Surgery, University of Bellevue Hospital Medical College, New York. Large quarto volume of 187 pages, containing 25 figures on 22 colored lithographic plates, and 89 text-cuts, 60 in colors. Philadelphia and London: W. B. Saunders & Co., 1905. Cloth, $5.50 net.

In the preparation of this book Professor Schultze had in mind the need of a work that would combine the features of a text-book with the educational advantages of an atlas. He has produced a work of great merit, and not alone the anatomist, but more practically the general practitioner will find it of constant value. Professor Schultze has presented his own methods for the study of anatomy—methods proved to be correct and practical by many years of clinical study. Throughout the work the value of the knowledge of topographic anatomy in bedside diagnosis is emphasized. The many colored lithographic plates and the numerous text-cuts, sixty of which are in colors, are of exceptional excellence. Indeed, both for accuratenesss of detail and artistic beauty, we have never seen their equal. The greater portion of the dissections from which these illustrations have been made are from the author's own preparations. Dr. George D. Stewart in editing the work has added many valuable notes.

CONSERVATIVE GYNECOLOGY AND ELECTRO-THERAPEUTICS. — A Practical Treatise on the Diseases of Women and Their Treatment by Eectricity. B. G. Betton Massey, M. D., Attending Surgeon to the American Oncologic Hospital, Philadelphia; Fellow and Ex-President of the American Electro-Therapeutic Association; Member of the Société Francaise d'Electro-Thérapie, American Medical Association, etc. Fourth edition, revised, rewritten and greatly enlarged. Illustrated with twelve (12) original, full-page chromo-lithographic plates; twelve (12) full-page half-tone plates of photographs taken from nature, and 157 half-tone and photo-engravings in the text. Pages XVI-468. Royal octavo. Extra cloth, beveled edges. Price, $4, net. F. A. Davis Company, Publishers, 1914-16 Cherry Street, Philadelphia.

This, the fourth revised editioin of this valuable work, is practically a new book; so much of it has been rewritten and added to. It is really a treatise on the medical and surgical diseases of women, with special reference to the therapeutic use of electricity. Special attention has been given to fibroid tumors and their treatment by the Apostoli method, and to the author's method of treating cancer.

---

CHEMICAL AND MICROSCOPICAL DIAGNOSIS. By Francis Carter Wood, M. D., Adjunct Professor of Clinical Pathology, College of Physicians and Surgeons, Columbia University, New York; Pathologist to St. Luke's Hospital, New York. One hundred and eighty-eight illustrations in the text, and nine colored plates. New York and London: D. Appleton & Co, 1905. Cloth. 745 pages. Price, not given (about $5).

Chemical and microscopical examinations are now recognized as necessary to correct diagnosis by all progressive physicians. Old methods are no longer to be entirely relied upon, and there are many obsc[illegible]e diseases that can be diagnosed only by more exact methods. All insurance companies now require it. A reliable treatise on the subject by an expert, such as Professor Wood, will, therefore, be hailed with joy by active practitioners, especially by the younger generation of doctors who *must be* up-to-date. This is an excellent work and most timely and opportune.

ELEGY IN A JOURNAL CHURCH YARD. *Hic Jacet*: The *Texas Medical Gazette*. Simultaneously with the appearance of the *Texas State Journal of Medicine* the *Gazette* "turned turtle" and "chucked it." What was it the coon said to Davy Crockett? "Don't shoot, I'll come down."

"The octopus tolls the knell of smaller fries;
Its wily tentacles creep slowly t'wards the sea;
The *Gazette* turns up its little toes and dies,
And leaves the field to *Matthew and to me."

---

THE TEXAS STATE JOURNAL OF MEDICINE. We have received (July 14) the initial number of the State Medical Association's new journal. It is a quarto of sixty pages of which sixteen are devoted to advertisements and twelve to a list of the members. The remaining thirty-two pages contain a part of the proceedings of the association at Houston, several excellent papers read at the meeting, correspondence, news of local society meetings, editorial matter, etc. It is a very creditable production and the association and its accomplished and enterprising young editor are to be congratulated upon it. The Red Back heartily wishes it success. It is no small credit to Austin that this work was awarded to the great publishing house of Von Boeckmann-Jones Co. on competitive bid. They published the transactions nearly twenty years. The publication was gotten out here and shipped to Fort Worth to be mailed. We hope Dr. Chase and his associates will be able to keep the *Journal of Medicine* up to the excellent standard inaugurated. We note in this connection with regret the suspension of the Fort Worth *Medical Gazette*. We shall miss it.

*Matthew M. Smith, The Texas Medical News.

